# miR-155 acts as an anti-inflammatory factor in atherosclerosis-associated foam cell formation by repressing calcium-regulated heat stable protein 1

**DOI:** 10.1038/srep21789

**Published:** 2016-02-22

**Authors:** Xiaoyi Li, Deyong Kong, Heming Chen, Shuiyi Liu, Hui Hu, Tangwei Wu, Jing Wang, Weiqun Chen, Yong Ning, Yong Li, Zhongxin Lu

**Affiliations:** 1 Department of Medical Laboratory, Central Hospital of Wuhan, Wuhan 430014, China; 2 School of Laboratory Medicine, Hubei University of Chinese Medicine, Wuhan 430065, China; 3 Department of Cardiovascular and Thoracic Surgery, Second Xiangya Hospital, Central South University, Changsha 410011, China; 4 Cancer Research Institute of Wuhan, Wuhan 430014, China; 5 Department of Central Laboratory, Central Hospital of Wuhan, Wuhan 430014, China; 6 Department of Cancer Biology, Lerner Research Institute, Cleveland Clinic, Cleveland, OH 44195, USA

## Abstract

Atherosclerosis (AS) is chronic inflammation in response to lipid accumulation. MicroRNA-155 (miR-155) is being increasingly studied to evaluate its potential as diagnostic biomarkers and therapeutic targets in many diseases. However, delineating the role of miR-155 in AS remains difficult. Here, we detected constitutive expression of several microRNAs (miRNAs) possibly associated with cardiovascular disease in foam cells and clinical specimens from patients with AS. Among them, we found that the level of miR-155 in foam cells was the most significantly elevated in a dose- and time-dependent manner. In addition, the expression of miR-155 was elevated in the plasma and plaque of patients with AS. We also reported for the first time that miR-155 targets *calcium-regulated heat stable protein 1* (*CARHSP1*), which regulates the stability of tumor necrosis factor alpha (TNF-α) mRNA. Furthermore, we investigated the mechanism by which the miR-155 level is elevated. miR-155 upregulation is due to transcriptional regulation by nuclear factor (NF)-κB, which is activated by the inflammatory factor TNF-α. In summary, increased miR-155 relieves chronic inflammation by a negative feedback loop and plays a protective role during atherosclerosis-associated foam cell formation by signaling through the miR-155–CARHSP1–TNF-α pathway.

AS is one of the most common cardiovascular diseases, leading to severe cardiovascular complications^1^. This disease is chronic inflammation in response to lipid accumulation, and macrophages are the main effector cells that stimulate the vascular inflammatory reaction throughout the pathological process^2^,^3^,^4^. The role of cholesterol loading in macrophage inflammation raises the broader issue of mechanisms and consequences of macrophage-mediated inflammation in AS^5^. In the early stages of AS, monocytes respond to inflammatory factors and oxidative stress and subsequently differentiate into macrophages^6^,^7^. In this process, macrophages are recruited to take up oxLDL to form lipid-overloaded foam cells^8^, which play a crucial role in the formation of atherosclerotic plaque and the progression of AS.

miRNAs are endogenous, noncoding, single-stranded RNAs, ~22 nucleotides in length which silence gene expression by binding to the 3′-UTR of target mRNAs, thereby suppressing their translation or promoting their degradation^9^,^10^,^11^. miRNA sequences are highly conserved between different species^12^. Recent studies suggest that these molecules are closely involved in the pathogenesis and progression of cardiovascular disease and are good diagnostic or prognostic biomarkers and therapeutic targets^13^,^14^. In this research, we focused on miR-155, which is encoded by the MIR155 host gene (*MIR155HG*) and transcribed from the B-cell integration cluster (BIC), located on chromosome 21^15^. Various reports have indicated that miR-155 is a typical multi-functional miRNA that is closely linked to several important inflammation diseases, including AS^16^,^17^. This molecule has an anti-inflammatory effect and plays a part in the prevention of AS development and progression by targeting MAP3K10 in macrophages^18^. On the other hand, miR-155 may promote AS, as it has been observed to derepress BCL6-mediated inhibition of CCL2 transcription in the bone marrow cells of *ApoE*^–/–^ mice^19^. These investigations have therefore demonstrated that miR-155 has opposite effects in different cell types, pathological stages, and animal models of AS.

CARHSP1, also known as CRHSP-24, is a cytoplasmic protein located in processing bodies or exosome granules and has been identified as a cold shock domain (CSD) protein family member. These proteins are composed of an evolutionarily conserved CSD, which functions as a transcriptional or translational regulator^20^,^21^. CARHSP1 contains a CSD, making it possible for it to bind to polypyrimidine regions and regulate the stability of single-stranded RNA or DNA^22^,^23^. CARHSP1 was first identified as a physiological substrate for the Ca^2+^/calmodulin-regulated protein phosphatase calcineurin (PP2B). Recently, it was found to be an mRNA stability enhancer for tumor necrosis factor alpha (TNF-α), which is the central mediator of inflammation in macrophages, and several studies have demonstrated that CARHSP1 binds to the AU-rich element (ARE) of the TNF-α 3′-UTR through the CSD^24^,^25^,^26^. The ARE binding site is critical for the regulation of mRNA translation, degradation and stability, and therefore these studies indicate that CARHSP1 is critical for TNF-α mRNA stabilization and has a significant role in the regulation of inflammation.

In this study, we found that miR-155 is overexpressed in the serum and atherosclerotic lesions of AS patients and that expression of miR-155 in oxLDL-treated THP-1 inflammatory macrophages was increased in a dose- and time-dependent manner. We also demonstrated that miR-155 inhibits CARHSP1, which affects the stability of TNF-α mRNA, thus involving this cytokine in the process of AS. Furthermore, we confirmed that the transcription factor NF-κB, which is activated by TNF-α, binds to the promoter of miR-155 and stimulates the transcription of miR-155. In summary, we determined that an increased miR-155 level relieves chronic inflammation and plays a protective role by signaling through the miR-155–CARHSP1–TNF-α pathway.

## Results

### miR-155 is overexpressed in the plasma and atherosclerotic lesions of patients with AS and is induced by oxLDL in human macrophages

**Figure 1:**
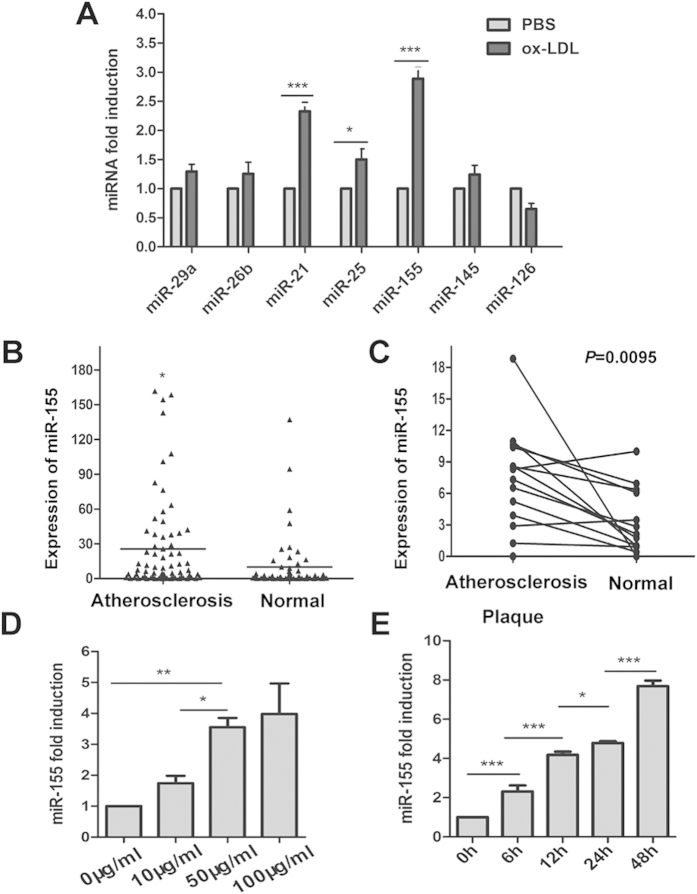
The expression of miR-155 is increased in human macrophages in clinical specimens and is induced by oxLDL. (**A**) The expression of miRNAs in oxLDL (50 μg/ml)-treated macrophages was determined by qRT-PCR. Phosphate-buffered saline was used as control. (**B**) Levels of miR-155 in plasma from patients with AS (n = 70) and normal controls (n = 55). Values were normalized to U6. In this dot plot, the horizontal line indicates the mean. (**C**) qRT-PCR assay of miR-155 in atherosclerotic lesions (n = 17) and normal veins (n = 17) from the same patients with AS (*P = *0.0095). (**D**) qRT-PCR analysis of miR-155 expression in THP-1 cells, which were first stimulated with propylene glycol monomethyl ether acetate (PMA, 100 nM) to induce them to differentiate into macrophages, then treated with oxLDL at the indicated doses (0, 10, 50, or 100 μg/ml). (**E**) qRT-PCR analysis of miR-155 expression in THP-1 cells, which were first stimulated with PMA (100 nM) to induce them to differentiate into macrophages, then treated with oxLDL (50 μg/ml) for the indicated times (0, 6, 12, 24, or 48 h). *P < 0.05, **P < 0.01, ***P < 0.001. Results are presented as mean ± SD of 3 independent experiments as determined by Bonferroni’s multiple comparison test after one-way ANOVA (**D,E**) or Student’s t test (**A–C**).

To identify novel miRNAs that are involved in atherogenesis, we established a foam cell model by inducing THP-1 cells with oxLDL and detected seven miRNAs possibly associated with cardiovascular disease by qRT-PCR. Among these miRNAs, miR-155 was the most significantly elevated in oxLDL-treated THP-1 cells (Fig. 1A). To confirm that the expression of miR-155 was increased in patients with AS, we measured the level of miR-155 in the plasma of patients with AS and in the normal control group, and the results showed that the miR-155 level was indeed upregulated in patients with AS (Fig. 1B). The relative expression level of miR-155 in 17 pairs of atherosclerotic lesions and normal veins from the same patients were also detected by qRT-PCR, and the results showed that the miR-155 level was significantly increased in the atherosclerotic lesions compared with the normal veins (Fig. 1C). To determine whether overexpression of miR-155 is induced by oxLDL, THP-1 cells were first treated with 100 nM PMA, then stimulated with oxLDL at the indicated concentrations (10, 50, and 100 μg/ml) for 24 h or with 50 μg/ml oxLDL for specific times (0, 6, 12, 24, and 48 h), while control cells were treated with PBS^27^. The results showed that the expression of miR-155 is increased by oxLDL stimulation in a dose- and time-dependent manner (Fig. 1D,E).

### miR-155 attenuates lipid uptake and suppresses the inflammatory response by repressing the expression of TNF-α in foam cell formation

**Figure 2:**
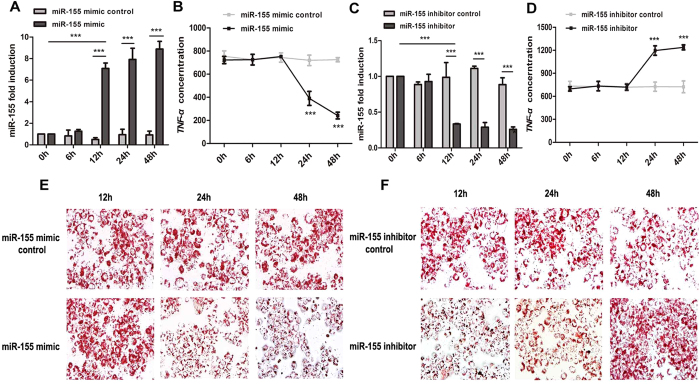
The role of miR-155 in countering inflammation and lipid uptake by repressing the expression of TNF-α. (**A,C**) The expression of miR-155 was examined by qRT-PCR for different times (0, 6, 12, 24, 48 h) following transfection of a miR-155 mimic, inhibitor, and control (100 nM) into macrophages that had been previously differentiated from THP-1 cells. (**B,D**) After transfection of the miR-155 mimic, inhibitor, and control (100 nM) into macrophages, these cells were then treated with oxLDL (50 μg/ml) for 24 h, and the secreted protein concentration of TNF-α was determined by ELISA. (**E,F**) Lipid uptake was measured by Oil Red O staining after transfection of the miR-155 mimic, inhibitor, and control (100 nM) into macrophages, then treated with oxLDL (50 μg/ml) for 24 h (magnification 400 × ). ***P < 0.001. Results are presented as mean ± SD of 3 independent experiments as determined by Student’s t test (**A–D**).

To investigate the role of miR-155 in the inflammatory response of AS, the macrophages that were stimulated by 100 nM PMA-treated THP-1 cells were transfected with 100 nM chemically synthesized miR-155 mimic or miR-155 inhibitor for 0, 6, 12, 24, or 48 hours, followed by treatment with 50 μg/ml oxLDL for 24 hours. The effect of miR-155 overexpression and knockdown on foam cell formation was analyzed by Oil Red O staining, and inflammatory factor secretion was examined by ELISA. The transfection efficiency of miR-155 mimic or inhibitor was examined by qRT-PCR (Fig. 2A,C). When cells were transfected with miR-155 mimic for 0, 6, or 12 hours, the secretion of TNF-α was invariant and similar to the control group. After transfection with miR-155 mimic for 24 or 48 hours, the secretion of TNF-α was significantly suppressed (Fig. 2B). By contrast, when cells were transfected with miR-155 inhibitor for 24 or 48 hours, the secretion of TNF-α increased (Fig. 2D). Furthermore, to determine the effect of miR-155 on foam cell formation, Oil Red O staining showed that, after transfection with miR-155 mimic for 12, 24, or 48 hours, lipid uptake was remarkably attenuated (Fig. 2E). By contrast, transfection with miR-155 inhibitor led to the inverse outcome of enhanced lipid uptake (Fig. 2F).

### CARHSP1 is required for TNF-α mRNA stabilization

**Figure 3:**
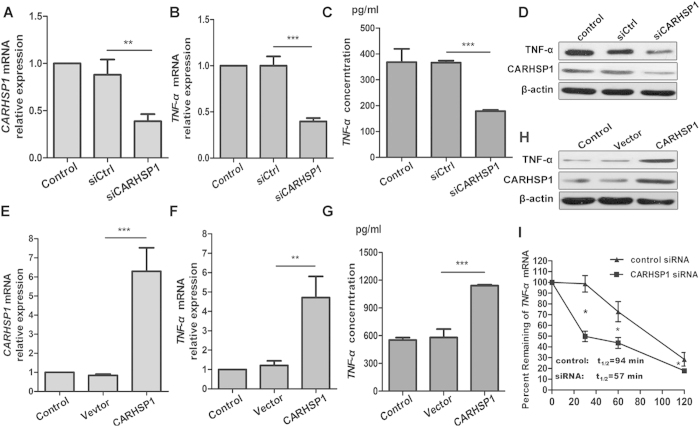
CARHSP1 is required for TNF-α mRNA stabilization. (**A,E**) qRT-PCR analysis of CARHSP1 expression to examine the efficiency of overexpression and knockdown. Overexpression group was transfected with pCMV6-AC plasmid (Vector, 1 μg), pCMV6-AC-CARHSP1 plasmid (CARHSP1, 1 μg). Knockdown group was transfected with CARHSP1 siRNA (siCARHSP1, 50 pmol/ml), siRNA control (siCtrl, 50 pmol/ml) for 24 h. The control group was a mock transfection. (**B,F**) qRT-PCR analysis of TNF-α mRNA expression to determine the effect of CARHSP1 overexpression or knockdown on the TNF-α mRNA level. (**C,G**) The secreted protein concentration of TNF-α was determined by ELISA to determine the effect of CARHSP1 overexpression or knockdown on the TNF-α secreted protein level. (**D,H**) The intracellular protein concentration of TNF-α was determined by western blot to determine the effect of CARHSP1 overexpression or knockdown on TNF-α intracellular protein level, with β-actin as a control. (**I**) TNF-αmRNA half-life (t_1/2_) in cells treated with actinomycin D (ActD) post transfection with CARHSP1 siRNA or the control. **P < 0.01, ***P < 0.001. Results are presented as mean ± SD of 3 independent experiments as determined by Bonferroni’s multiple comparison test after one-way ANOVA (**A–C,E–G**) and RNA half-life (t_1/2_) was calculated by linear regression analysis (**I**). Full-length blots are presented in Supplementary Figures S1 and S2.

CARHSP1 was previously identified as a TNF-α 3′-UTR-interacting protein by luciferase assays and mRNA stability assays after CARHSP1 overexpression or CARHSP1 knockdown^26^,^28^. To verify that CARHSP1 is required for TNF-α mRNA stabilization in foam cells, we transfected siRNA control or CARHSP1 siRNA into THP-1 cells, and the cells were then treated with oxLDL to form foam cells. qRT-PCR and western blotting were then used to measure the effect of CARHSP1 knockdown on TNF-α mRNA and protein. The transfection efficiency of CARHSP1 siRNA was examined by qRT-PCR (Fig. 3A) and western blotting (Fig. 3D), and the results indicate that CARHSP1 knockdown down-regulates TNF-α protein production and reduces the level of TNF-α mRNA (Fig. 3B–D). Additionally, we cloned the CARHSP1 ORF into the pCMV6-AC-GFP vector to overexpress CARHSP1 and transfected it into THP-1 cells, and the transfection efficiency of the CARHSP1 overexpression plasmid was examined by qRT-PCR (Fig. 3E). The results showed that CARHSP1 overexpression increases the level of TNF-α mRNA and up-regulates TNF-α protein production (Fig. 3F–H). We also validated the role of CARHSP1 in TNF-α regulation using an mRNA stability assay. The TNF-αmRNA half-life was 94 min in cells with the siRNA control and 57 min in the CARHSP1 knockdown cells (Fig. 3I). These results suggest that CARHSP1 is required for TNF-α mRNA stabilization.

### CARHSP1 is a functional target of miR-155

**Figure 4:**
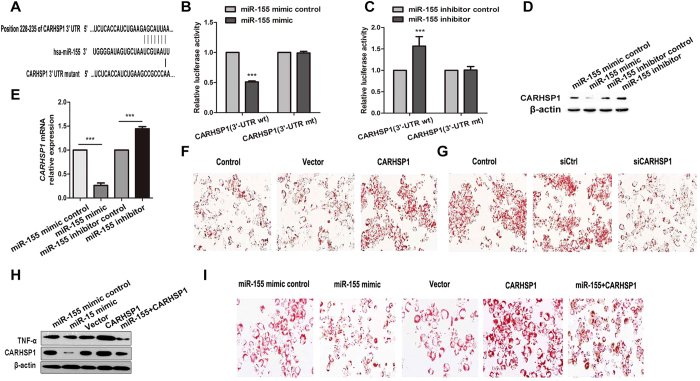
Validation of CARHSP1 as a functional target of miR-155. (**A**) The predicted binding site of miR-155 in the 3′-UTR of *CARHSP1* is indicated. A *CARHSP1* 3′-UTR mutant with a mutation in the miR-155 binding site is also shown. (**B,C**) After co-transfection with the pRL-TK plasmid carrying a wild type or mutant 3′-UTR sequence and a miR-155 mimic (10 pmol) or a miR-155 inhibitor (10 pmol) into HEK-293T cells for 48 h, the luciferase activity was measured. (**D,E**) The protein and mRNA level of CARHSP1 was measured by western blot and qRT-PCR after transfection with the miR-155 mimic (100 nM), the miR-155 inhibitor (100 nM), the mimic control, or the inhibitor control into THP-1 cells at 48 h. (**F,G**) Lipid uptake by foam cells was detected by Oil Red O staining after transfection of the pCMV6-AC-CARHSP1 plasmid (1 μg), CARHSP1 siRNA (siCARHSP1, 50 pmol/ml), plasmid control, or siRNA control (siCtrl) for 24 h in macrophages, which were treated with oxLDL for 24 h (magnification, 400 × ). (**H,I**) Western blot analysis and Oil Red O staining were employed to examine the protein level of CARHSP1 and TNF-α after transfection with miR-155 mimic control, miR-155 mimic, pCMV6-AC-GFP (defined as Vector), or pCMV6-AC-CARHSP1 or co-transfection with miR-155 mimic and pCMV6-AC-CARHSP1, with β-actin as western blot control. ***P < 0.001. Results are presented as mean ± SD of 3 independent experiments as determined by performing a Student’s t test (**B,C,E**). Full-length blots are presented in Supplementary Figures S4 and S5.

To elucidate the mechanism by which miR-155 affects macrophage-derived foam cell formation and the inflammatory response, we performed several computational analyses available in online miRNA target databases, including MirDB, Targetscan, and PicTar, to identify potential direct mRNA targets of miR-155. Among many predicted target genes of miR-155 in miRBase, CARHSP1 (also known as CRHSP-24), which acts as an mRNA stability enhancer for TNF-α, is a newfound target for miR-155 and has a binding site for miR-155. The binding sites to the miR-155 seed sequence are highly conserved in human, mouse, rat, rabbit, and horse (Figure S3). To validate the predicted miRNA binding site, we performed a luciferase reporter assay in HEK293T cells by transfecting luciferase reporters with either the wild-type CARHSP1 3′-UTR or a mutant lacking the miR-155 binding site (Fig. 4A). We found that transfection with the miR-155 mimic and a CARHSP1 3′-UTR reporter vector resulted in a significant reduction of luciferase activity (Fig. 4B). By contrast, transfection with the miR-155 inhibitor caused a significant increase in luciferase activity (Fig. 4C).

To identify the potential relationship between miR-155 and CARHSP1, qRT-PCR was employed to measure the mRNA levels of CARHSP1 in oxLDL-treated macrophages with or without miR-155 involvement. The cytoplasmic protein was collected from the cells, which were transfected with miR-control, miR-155 mimic, or miR-155 inhibitor for 24 h and subsequently stimulated with oxLDL for 24 h to determine whether miR-155 affects translation of CARHSP1. Western blot results revealed that overexpressed miR-155 also inhibits CARHSP1 protein expression (Fig. 4D). Furthermore, the qRT-PCR results indicate that the mRNA levels of CARHSP1 are downregulated by overexpressed miR-155 (Fig. 4E).

To further examine whether CARHSP1 is a functional target of miR-155 during foam cell formation, THP-1 cells were transfected with miR-155 mimic, CARHSP1 siRNA, or a CARHSP1 overexpression vector, and it was found that overexpression of CARHSP1 increased the protein level of TNF-α and lipid uptake in oxLDL-induced foam cells (Figs 3H and 4F), while knockdown of CARHSP1 significantly decreased the protein level of TNF-α and strongly prevented lipid uptake, as shown by Oil Red O staining (Figs 3D and 4G). Moreover, overexpression of CARHSP1 rescued the protein level of CARHSP1 that had been decreased by overexpressed miR-155 (Fig. 4H) and reversed the protective effects caused by overexpression of miR-155 on lipid uptake in oxLDL-induced foam cells (Fig. 4I). All these results indicate that CARHSP1 is a functional target of miR-155, which directly targets its 3′UTR, and that CARHSP1 serves as a mediator of miR-155 by regulating the production of TNF-α and thereby affecting foam cell formation.

### Overexpression of miR-155 is induced by NF-κB, which is activated by TNF-α

**Figure 5:**
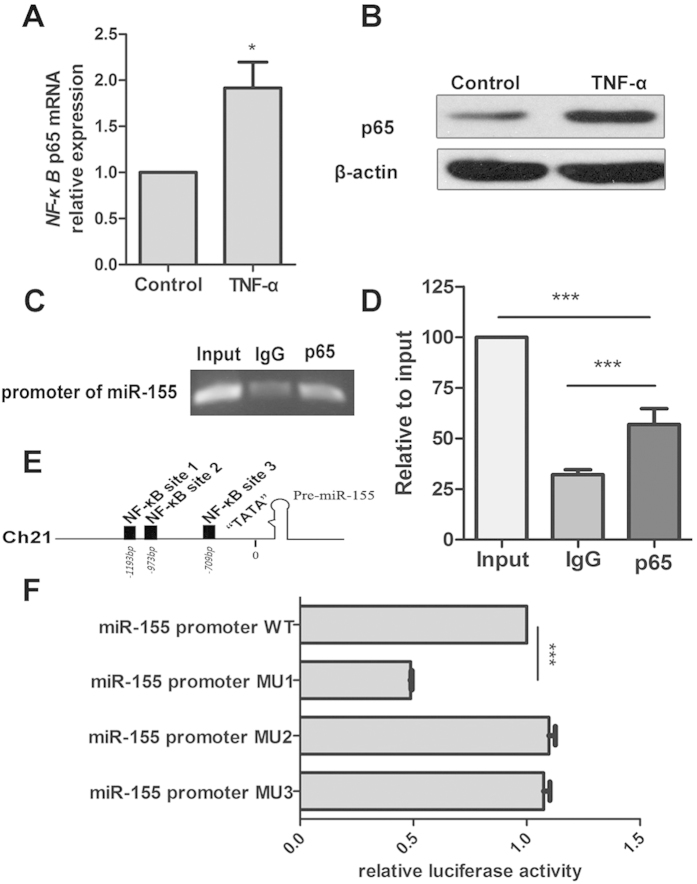
NF-κB p65 is activated by TNF-α and subsequently induces transcription of miR-155. (**A,B**) The mRNA and protein levels of NF-κB p65 were measured by qRT-PCR and western blot after treatment with TNF-α (20 ng/ml) for 6 h, with phosphate-buffered saline used as control. (**C,D**) A ChIP assay was performed with nuclear extracts of macrophages, which were differentiated from THP-1 cells treated with TNF-α (20 ng/ml) for 6 h using a specific p65 antibody. The ChIP assay input served as a positive control, and normal IgG was used as a negative control. (**E**) Three potential binding sites (referred to as sites 1, 2, and 3) for NF-κB in the promoter region of pri-miR-155 were located in the region (–2000 to –1 bp) upstream of pri-miR-155/BIC. (**F**) Luciferase activity measurements in macrophages (differentiated from THP-1 cells) treated with TNF-α (20 ng/ml) for 6 h and transfected with the pRL-TK-pri-miR-155-luciferase wild type reporter or three pRL-TK-pri-miR-155-luciferase mutant reporters for 24 h. ***P < 0.001. Results are presented as mean ± SD of 3 independent experiments as determined by performing Bonferroni’s multiple comparison test after one-way ANOVA (**D,F**) or Student’s t test (**A**). Full-length blots/gels are presented in Supplementary Figures S6 and S7.

Previous studies have demonstrated that TNF-α activates NF-kB^29^,^30^. To investigate how miR-155 is induced during foam cell formation, THP-1 cells were stimulated by TNF-α for 6 hours. We measured the mRNA and protein levels of NF-κB to confirm that endogenous NF-kB is induced by TNF-α at the indicated concentrations and times (Fig. 5A,B). Next, a ChIP assay was performed to determine whether NF-κB directly binds to the pri-miR-155 genomic locus, and results showed that this was the case (Fig. 5C,D). Moreover, using the TRANSFAC database tool, we found that there were three potential binding sites (referred to as sites 1, 2, and 3) for NF-κB in the promoter region of pri-miR-155, which were located in the region (−2000 to −1 bp) upstream of pri-miR-155/BIC (Fig. 5E). Next, wild type or each of three different mutant reporter plasmids were transfected into TNF-α-treated THP-1 cells, and the luciferase results showed that site 1 was the strongest binding site (Fig. 5F). All these results suggest that NF-κB, which is activated by TNF-α in THP-1 cells, is a transcriptional enhancer of miR-155.

## Discussion

Chronic inflammation driven by cholesterol accumulation in macrophages is instrumental in atherosclerotic lesion formation. It is also known that macrophage exposure to oxLDL leads to formation of lipid-laden foam cells^31^. In this study, we found that oxLDL-induced miR-155 plays a crucial role in the development of AS by regulating foam cell formation. We showed that miR-155 functions as an anti-inflammatory rather than pro-inflammatory factor in foam cells at the advanced stages of the atherosclerotic condition. The expression of miR-155 is critical for the production of TNF-α by directly targeting CARHSP1, which was previously shown to be required for the stabilization of TNF-α mRNA^26^. The absence of miR-155 in macrophages induced an inflammation reaction and foam cell formation by upregulating CARHSP1. Moreover, we found that it was NF-κB which was activated by TNF-α that bound to the promoter of miR-155 and positively regulated the expression of miR-155. Therefore, all the results suggest that increased miR-155 levels relieve chronic inflammation and foam cell formation by signaling through the miR-155–CARHSP1–TNF-α pathway.

Many researchers have demonstrated that miRNAs play a vital role in the pathogenesis of AS. Recently, miR-126-5p was discovered to maintain a proliferative reserve in endothelial cells (ECs) through repression of the Notch1 inhibitor delta-like 1 homolog (Dlk1), thereby preventing atherosclerotic lesion formation^32^. Similarly, another group reported that miR-223 was transported to ECs in high-density lipoprotein (HDL) and decreased expression of ICAM-1 in ECs, indicating that the anti-inflammatory function of HDL is conferred through miRNA^33^. By contrast, Menghini *et al.* reported that miR-217 was expressed in human atherosclerotic lesions and is negatively correlated with silent information regulator 1 (Sirt1), resulting in endothelial senescence^34^. Interestingly, certain miRNAs, including miR-155, have contradictory effects on the pathogenesis of AS. Most reports have supported that miR-155 acts as an anti-inflammatory, atheroprotective microRNA^18^,^27^,^35^,^36^,^37^,^38^,^39^. Yet several studies reported a pro-atherogenic role for this microRNA^19^,^40^,^41^. We believe that the function of miR-155 is highly dependent on the context and cell types because of its dynamic expression pattern and its suppression of multiple target genes. Zhang *et al.* use carotid artery ligation model to assess the effect of Tongxinluo (TXL), a traditional Chinese medicine, exerts its inhibitory action on neointima formation by suppressing miR-155^41^. Tian *et al.* use macrophages from mice and Raw264.7 mouse cells to detect lipid uptake^42^. We use a classic human foam cell model *in vitro* to study the function of miR-155 in AS. Most likely, there is a specie-specific role of miR-155 in AS.

CARHSP1 was originally identified in pancreatic acinar cells by ^32^P metabolic labeling^43^, and a group has speculated that CARHSP1 participates in the oxidative stress response via a dynamic and temporal association between stress granules and processing bodies^44^. Although a recent study has demonstrated that CARHSP1 is a TNF-α mRNA stability enhancer required for effective TNF-α production and the importance of both stabilization and destabilization pathways in regulating TNF-α mRNA half-life^26^, the biological effects of CARHSP1 have remained elusive. Here we found for the first time that miR-155 targets the 3′-UTR of CARHSP1 and inhibits CARHSP1 at the mRNA and protein level, thus indirectly down-regulating the expression of TNF-α. We also found that down-regulation of TNF-α attenuates the inflammation reaction and lipid uptake by macrophages. We therefore speculated that CARHSP1 is a functional target of miR-155, which directly targets its 3′-UTR, and that CARHSP1 serves as a mediator of miR-155 by regulating the production of TNF-α and thereby affecting foam cell formation.

After investigating the biological effects of miR-155 and the relationship with its target, we were also interested in the question of how miR-155 is upregulated during the pathological process of AS. Recent studies have shown that several transcription factors, including AP-1, STAT3, and MYB, up-regulate the expression of miR-155 in the immune system^45^,^46^,^47^. Since TNF-α is overexpressed at the early stages of foam cell formation, we hypothesized that NF-κB is activated by up-regulated TNF-α, and bioinformatic tools revealed that there were several binding sites in the region of the miR-155 promoter^29^,^30^. Our ChIP assay and luciferase results were previously supported by some researchers and showed that NF-κB, which is activated by TNF-α in THP-1 cells, is a transcriptional enhancer of miR-155^48^,^49^. We suggest that, at the initial stages of foam cell formation, inflammatory factors such as TNF-α are overexpressed, and NF-κB is then activated by up-regulated TNF-α, thus resulting in a sharp increase in the miR-155 level. Based on our data, it appears that at the early stage of AS, the infiltrating monocytes are stimulated by inflammatory factors like TNF-α and differentiate into macrophages; due to the hyperlipidemia and oxidative modification, macrophages uptake oxLDL and differentiate into foam cells that deposit in endothelium, resulting atherosclerotic plaques. During the process, TNF-α activates NF-κB to transactivate the expression of miR-155, which in turn suppresses TNF-α production through directly targeting the 3′-UTR of CARHSP1. Therefore, miR-155 is a key component of a negative feedback loop to prevent atherosclerosis-associated foam cell formation by repressing CARHSP1.

A notable limitation of this study is that the importance of the miR-155-CARHSP1 axis in atherosclerosis needs to be reinforced by transgenic animal data. Another limitation is that beyond macrophages there are other types of cells contributing to the elevated plasma miR-155 levels in AS patients.

To summarize, we found that miR-155 is overexpressed in the plasma and atherosclerotic lesions of patients with AS, and its induction by oxLDL in human macrophages relieves inflammation and foam cell formation by depressing CARHSP1-mediated stimulation of TNF-α expression. We therefore conclude that miR-155 plays a key role in the anti-inflammation activity of macrophages, which thereby attenuates atherosclerosis-associated foam cell formation.

## Methods

### Patient characteristics

We enrolled 70 patients with AS, who were diagnosed by clinical symptoms and angiography from the Central Hospital of Wuhan between January 2014 and February 2015. Fifty-five healthy volunteers were recruited as the control group. We also identified 17 pairs of atherosclerotic lesions and normal veins from the same patients. Patients were excluded who had the following: 1) Any disease that activates monocytes, such as arthritis, bronchial asthma, and infectious diseases caused by bacteria or viruses. 2) Any serious cardiovascular disease, such as acute myocardial infarction or heart failure. Informed consent was obtained from all human subjects. This study was approved by the Medical Ethics Committee of The Central Hospital of Wuhan and all human subject research was performed in accordance with institutional, national, and Declaration of Helsinki requirements.

### Plasma collection and storage

Peripheral blood plasma from patients and the control group were collected in EDTA tubes and processed within 4 hours by centrifuging at 1000 *x g* at 4 °C for 10 minutes, gently transferring the plasma to a fresh RNase/DNase-free 1.5-mL EP tube (Axygen, Union City, CA, USA) while avoiding pipetting of the middle layer of white blood cells, and centrifuging again at 16,000 *x g* at 4 °C for 10 minutes. The supernatant was then transferred to fresh RNase/DNase-free tubes and stored at −80 °C.

### Cell culture and establishing the foam cell model

The human monocytic cell line THP-1 and human embryonic kidney (HEK)293T cells were purchased from the American Type Culture Collection (ATCC). THP-1 cells were cultured in RMPI-1640 medium (Gibco, Carlsbad, CA, USA), and HEK-293T cells were cultured in Dulbecco’s modified Eagle medium (DMEM, Gibco). Both media were supplied with 10% fetal bovine serum (Gibco), 100 μg/mL streptomycin, and 100 IU/mL penicillin (Thermo Fisher Scientific, Rockford, IL, USA). Cells were incubated in a humidified incubator at 37 °C with 5% CO_2_. To drive monocyte differentiation into macrophages and ultimately establish a foam cell model, THP-1 cells were first seeded at 1 × 10^6^ per mL with 100 nM propylene glycol monomethyl ether acetate (PMA, Sigma-Aldrich, St. Louis, MO, USA) for 12 hours to differentiate them into adherent macrophages^18^,^50^. The THP-1 macrophages were then stimulated with 50 μg/ml oxLDL (Luwen, Shanghai, China) for 12 hours to establish the foam cell model^51^.

### Oil Red O staining

Macrophages derived from THP-1 cells were transfected with a miR-155 mimic or a miR-155 inhibitor at a working concentration of 100 nM using Lipofectamine^®^ LTX and Plus reagent (Invitrogen, Carlsbad, CA, USA). After 24 h, the cells were washed three times with 1 × phosphate-buffered saline (PBS), fixed with 4% paraformaldehyde for 30 minutes, then stained with Oil Red O stain (Beisuo, Zhuhai, Shenzhen, China) in a 6:4 ratio of 0.5% Oil Red O solution with ddH_2_O for 20 minutes, followed by destaining with 60% isopropanol for 1 minute. The foam cells were then photographed under a microscope (Olympus, Tokyo, Japan) at 400 × magnification.

### RNA isolation and qRT-PCR

Plasma miRNA from AS patients and healthy volunteers was extracted using the mirVana PARIS kit (Ambion, Carlsbad, CA, USA) according to the manufacturer’s instructions. miRNA reverse transcription was performed using the TaqMan MicroRNA Reverse Transcription kit (Applied Biosystems, Carlsbad, CA, USA), and the miRNAs were analyzed by qRT-PCR using the TaqMan Small RNA Assay kit and TaqMan universal PCR master mix (Applied BioSystems) in an ABI StepOne Plus qPCR instrument (Applied Biosystems). Total RNA from THP-1 macrophages and atherosclerotic lesions was isolated using TRIzol reagent (Invitrogen). Total RNA was measured using SYBR Green (Applied BioSystems) in an ABI StepOne Plus qPCR instrument, and cDNA for target gene detection was synthesized from 1 μg total RNA. The following primers were used: CARHSP1 sense (5′-CCTGCACATCTCTGATGTGGA-3′) and antisense (5′-TGGTGCCAGGTGAGTGATGA-3′); TNF-α sense (5′-GTAGCCCATGTTGTAGCAAACCCTC-3′) and antisense (5′TGAAGAGGACCTGGGAGTAGAT3′); NF-κB p65 sense (5′-TCCCATCTTTGACAATCGTGCCC-3′)and antisense (5′-TCTGCACCTTGTCACACAGTAG-3′). PCR results were normalized between samples with U6 as an internal control and expressed as 2^–(CT[miRNA]–CT[U6])^, with CT denoting the threshold cycle^52^.

### Western blot

Cells were lysed in RIPA buffer containing protease inhibitors (Beyotime, Shanghai, China), and the protein concentration was measured using a BCA protein assay kit (Thermo Fisher Scientific). Soluble lysate was mixed with loading buffer and boiled for 10 min. Lysates were separated via 12% SDS-PAGE electrophoresis, then transferred to a 0.22-μm PVDF membrane and blocked with 5% nonfat milk. The membranes were then incubated with primary antibodies against β-actin (1:1000, Santa Cruz, Delaware, CA, USA), CARHSP1 (1:1000, Santa Cruz), or TNF-α (1:1000, R&D Systems, Minneapolis, MN, USA) overnight at 4 °C. The membranes were then incubated with the HRP-conjugated secondary antibody (1:10,000, Santa Cruz) and the protein level detected by chemiluminescence.

### Enzyme-linked immunosorbent assay (ELISA) for cytokine detection

Secreted pro-inflammatory cytokines (TNF-α) were detected in aliquots of THP-1 macrophage supernatants and quantitated by ELISA (R&D Systems). The entire procedure was performed according to the manufacturer’s instructions.

### Luciferase reporter assay

The prediction of miRNA target genes was performed using online software packages, including MirDB, Targetscan, and PicTar. A fragment of the CARHSP1 3′-UTR containing a putative miR-155 binding site was amplified from human genomic DNA from THP-1 cells by PCR using specific primers (sense, 5′-TATCTAGAGGAGGAGGCAGCAGACACT-3′; antisense, 5′-ATGCGGCCGCAAAGGCAACATCCGTCCAAT-3′). A mutant 3′-UTR lacking the miR-155 seed sequence was also amplified with specific primers (sense 5′-TATCTAGAGGAGGAGGCAGCAGACACT-3′; antisense, 5′-AATGCTTTTGGGCGGCTTCAGATGGTGAGAGG-3′). The amplified products were restricted and ligated to the XbaI and NotI (New England Biolabs, NEB, Ipswich, MA, England) sites of the pRL-TK vector (Promega, Madison, WI, USA), and the reporter plasmids were confirmed by DNA sequencing. Approximately 1 × 10^4^ HEK-293T cells were seeded into 96-well plates 24 hours before transfection, and the cells were then co-transfected with pRL-TK-CARHSP1-3′UTR or pRL-TK-CARHSP1-3′UTR mutant vectors (100 ng), 10 ng of pGL3 control (Promega), and 10 pmol miR-155 mimic or miR-155 inhibitor using Lipofectamine^®^ LTX and Plus reagent (Invitrogen). After a 48-hour transfection, luciferase activity was detected using the Dual-Glo luciferase reporter assay system (Promega), according to the manufacturer’s protocol. miR-155 mimic and miR-155 inhibitor were purchased from GenePharma (Suzhou, Jiangsu, China). The mimic is a chemically synthesized double-stranded RNA oligonucleotide that mimics the effects of endogenous miR-155. The inhibitors are chemically synthesized miRNA hairpin inhibitors that effectively inhibit endogenous miR-155 function.

### Transfection of miR-155 promoter constructs and luciferase assay

The human miR-155 promoter region containing NF-κB p65 binding sites, extending from –1 bp to –2000 bp relative to the transcription start site, was generated by PCR using the following primers: sense, 5′-TATCTAGAGCGAGTTATATTGGCTGGGGTGG-3′; antisense, 5′-ATGCGGCCGC TGTGATACAAGCAATGGAGGT-3′. The genomic DNA of THP-1 cells was isolated for use as template. The purified fragment was digested using XbaI and NotI and inserted into the XbaI- and NotI-cut pRL-TK luciferase reporter vector. The promoter region was sequenced and compared with sequences in Genbank. The miR-155 promoters mutated at the p65 binding site 1 (–1108 bp to –1099 bp), site 2 (–978 bp to –969 bp), and site 3 (–714 bp to –704 bp) were generated by site-directed mutagenesis using a special polymerase contained in the Phusion^TM^ High-Fidelity PCR kit (NEB).

### Chromatin immunoprecipitation (ChIP) assay

To detect which putative binding site within the miR-155 promoter binds to NF-κB p65, chromatin immunoprecipitation assays were performed using the Magna ChIP™ HiSens kit (Millipore, Temecula, CA, USA), according to the manufacturer’s instructions. In brief, proteins and DNA were first cross-linked using 37% formaldehyde for 10 minutes, and the cells were then lysed in lysis buffer for 15 minutes to release cross-linked protein–DNA complexes. In the next step, the isolated chromatin was sonicated on wet ice to shear the DNA, yielding chromatin fragments of 200–1000 bp, and the final step was immunoprecipitation (IP) of cross-linked protein–DNA complexes using p65 antibody (4 μg, Millipore) after incubation overnight at 4 °C. The ChIP results were semi-quantified by agarose gel electrophoresis.

### CARHSP1 overexpression or knockdown

The open reading frame (ORF) sequence of CARHSP1 was searched using the UCSC and Ensemble genome browsers, and the ORF clone was amplified by PCR in order to append cloning sites to the 5′ and 3′ ends of the sequence using the following primers: sense, 5′-GAGGCGATCGCCATGTCATCTGAGCCTCCCCC-3′; antisense, 5′-GCGACGCGTGGAGCTGATGACATGTCCAGACCA-3′. The RNA was first isolated from THP-1 cells, and the full-length cDNA clone used as the template for CARHSP1 ORF cloning using the Phusion ^TM^ High-Fidelity PCR kit (NEB). The size of the amplification product was confirmed by agarose gel electrophoresis. The amplification product was digested using Sgf I and MIu I (NEB) and inserted into the Sgf I- and MIu I-cut pCMV6-AC-GFP vector (Origene, Rockville, MD, USA). The CARHSP1 overexpression plasmid sequences were confirmed by DNA sequencing, and the transfection efficiency was determined by qPCR. CARHSP1 siRNA was purchased from Santa Cruz Biotechnology, and 50 pmol/ml siRNA was transfected into THP-1 cells according to the manufacturer’s instructions.

### mRNA stability assay

OxLDL stimulated THP-1 cells were treated with actinomycin D (ActD) (5 μg/ml) to inhibit transcription after transfection with CARHSP1 siRNA or control. Total RNAs were extracted at different time points after ActD administration and TNF-α mRNA was measured by qRT-PCR. RNA half-life (t_1/2_) was calculated by linear regression analysis^53^,^54^.

### Statistical analysis

Student’s t-test was performed for comparison between two groups, and the data was also analyzed by one-way analysis of variance (ANOVA ) among multiple groups using GraphPad Prism software. All measurements were performed at least three times independently. *P* value < 0.05 were considered to be a statistically significant difference. *P < 0.05, **P < 0.01, ***P < 0.001.

## Additional Information

**How to cite this article**: Li, X. *et al.* miR-155 acts as an anti-inflammatory factor in atherosclerosis-associated foam cell formation by repressing calcium-regulated heat stable protein 1. *Sci. Rep.* **6**, 21789; doi: 10.1038/srep21789 (2016).

## Supplementary Material

Supplementary Information

## References

[b1] Glass C. K. & Witztum J. L. Atherosclerosis. the road ahead. Cell 104, 503–516 (2001). 11239408 10.1016/s0092-8674(01)00238-0

[b2] Moore K. J. & Tabas I. Macrophages in the pathogenesis of atherosclerosis. Cell 145, 341–355 (2011). 21529710 10.1016/j.cell.2011.04.005 PMC3111065

[b3] Tuttolomondo A. *et al.* Atherosclerosis as an inflammatory disease. Curr Pharm Des 18, 4266–4288 (2012). 22390643 10.2174/138161212802481237

[b4] Hansson G. K. Inflammation, atherosclerosis, and coronary artery disease. N Engl J Med 352, 1685–1695 (2005). 15843671 10.1056/NEJMra043430

[b5] Moore K. J. & Tabas I. Macrophages in the Pathogenesis of Atherosclerosis. Cell 145, 341–355 (2011). 21529710 10.1016/j.cell.2011.04.005 PMC3111065

[b6] Ross R. Atherosclerosis is an inflammatory disease. Am Heart J 138, S419–420 (1999). 10539839 10.1016/s0002-8703(99)70266-8

[b7] Weber C. & Noels H. Atherosclerosis: current pathogenesis and therapeutic options. Nat Med 17, 1410–1422 (2011). 22064431 10.1038/nm.2538

[b8] Ouimet M. & Marcel Y. L. Regulation of lipid droplet cholesterol efflux from macrophage foam cells. Arterioscler Thromb Vasc Biol 32, 575–581 (2012). 22207731 10.1161/ATVBAHA.111.240705

[b9] Ambros V. microRNAs: tiny regulators with great potential. Cell 107, 823–826 (2001). 11779458 10.1016/s0092-8674(01)00616-x

[b10] Williams A. E. Functional aspects of animal microRNAs. Cell Mol Life Sci 65, 545–562 (2008). 17965831 10.1007/s00018-007-7355-9 PMC11131689

[b11] Eulalio A., Huntzinger E. & Izaurralde E. Getting to the root of miRNA-mediated gene silencing. Cell 132, 9–14 (2008). 18191211 10.1016/j.cell.2007.12.024

[b12] Ambros V. The functions of animal microRNAs. Nature 431, 350–355 (2004). 15372042 10.1038/nature02871

[b13] Devaux Y. *et al.* MicroRNAs: new biomarkers and therapeutic targets after cardiac arrest? Crit Care 19, 54 (2015). 25886727 10.1186/s13054-015-0767-2 PMC4324045

[b14] Wang G. K. *et al.* Circulating microRNA: a novel potential biomarker for early diagnosis of acute myocardial infarction in humans. Eur Heart J 31, 659–666 (2010). 20159880 10.1093/eurheartj/ehq013

[b15] Eis P. S. *et al.* Accumulation of miR-155 and BIC RNA in human B cell lymphomas. Proc Natl Acad Sci USA 102, 3627–3632 (2005). 15738415 10.1073/pnas.0500613102 PMC552785

[b16] Faraoni I., Antonetti F. R., Cardone J. & Bonmassar E. miR-155 gene: a typical multifunctional microRNA. Biochim Biophys Acta 1792, 497–505 (2009). 19268705 10.1016/j.bbadis.2009.02.013

[b17] Ma X., Ma C. & Zheng X. MicroRNA-155 in the Pathogenesis of Atherosclerosis: A Conflicting Role? Heart, Lung and Circulation 22, 811–818 (2013). 10.1016/j.hlc.2013.05.651 23827206

[b18] Zhu J. *et al.* Regulation of microRNA-155 in atherosclerotic inflammatory responses by targeting MAP3K10. PLoS One 7, e46551 (2012). 23189122 10.1371/journal.pone.0046551 PMC3506618

[b19] Nazari-Jahantigh M. *et al.* MicroRNA-155 promotes atherosclerosis by repressing Bcl6 in macrophages. J Clin Invest 122, 4190–4202 (2012). 23041630 10.1172/JCI61716 PMC3484435

[b20] Wistow G. Cold shock and DNA binding. Nature 344, 823–824 (1990). 2184368 10.1038/344823c0

[b21] Lindquist J. A., Brandt S., Bernhardt A., Zhu C. & Mertens P. R. The role of cold shock domain proteins in inflammatory diseases. J Mol Med 92, 207–216 (2014). 24562821 10.1007/s00109-014-1136-3

[b22] Schafer C., Steffen H., Krzykowski K. J., Goke B. & Groblewski G. E., CRHSP-24 phosphorylation is regulated by multiple signaling pathways in pancreatic acinar cells. Am J Physiol Gastrointest Liver Physiol 285, G726–734 (2003). 12801884 10.1152/ajpgi.00111.2003

[b23] Hou H. *et al.* Structure-functional analyses of CRHSP-24 plasticity and dynamics in oxidative stress response. J Biol Chem 286, 9623–9635 (2011). 21177848 10.1074/jbc.M110.177436 PMC3058955

[b24] Yan G., You B., Chen S. P., Liao J. K. & Sun J. Tumor necrosis factor-alpha downregulates endothelial nitric oxide synthase mRNA stability via translation elongation factor 1-alpha 1. Circ Res 103, 591–597 (2008). 18688046 10.1161/CIRCRESAHA.108.173963 PMC2753820

[b25] Zhang T., Kruys V., Huez G. & Gueydan C. AU-rich element-mediated translational control: complexity and multiple activities of trans-activating factors. Biochem Soc Trans 30, 952–958 (2002). 12440953 10.1042/bst0300952

[b26] Pfeiffer J. R., McAvoy B. L., Fecteau R. E., Deleault K. M. & Brooks S. A. CARHSP1 Is Required for Effective Tumor Necrosis Factor Alpha mRNA Stabilization and Localizes to Processing Bodies and Exosomes. Mol Cell Biol 31, 277–286 (2010). 21078874 10.1128/MCB.00775-10 PMC3019981

[b27] Zhu G. *et al.* miR-155 inhibits oxidized low-density lipoprotein-induced apoptosis of RAW264.7 cells. Mol Cell Biochem 382, 253–261 (2013). 23797321 10.1007/s11010-013-1741-4

[b28] Lindquist J. A., Brandt S., Bernhardt A., Zhu C. & Mertens P. R. The role of cold shock domain proteins in inflammatory diseases. J Mol Med 92, 207–216 (2014). 24562821 10.1007/s00109-014-1136-3

[b29] De Simone V. *et al.* Th17-type cytokines, IL-6 and TNF-alpha synergistically activate STAT3 and NF-kB to promote colorectal cancer cell growth. Oncogene 34, 3493–3503 (2015). 25174402 10.1038/onc.2014.286 PMC4493653

[b30] Dvoriantchikova G. & Ivanov D. Tumor necrosis factor-alpha mediates activation of NF-κB and JNK signaling cascades in retinal ganglion cells and astrocytes in opposite ways. Eur J Neurosci 40, 3171–3178 (2014). 25160799 10.1111/ejn.12710 PMC4205188

[b31] Weber C. & Noels H. Atherosclerosis: current pathogenesis and therapeutic options. Nat Med 17, 1410–1422 (2011). 22064431 10.1038/nm.2538

[b32] Schober A. *et al.* MicroRNA-126-5p promotes endothelial proliferation and limits atherosclerosis by suppressing Dlk1. Nat Med 20, 368–376 (2014). 24584117 10.1038/nm.3487 PMC4398028

[b33] Tabet F. *et al.* HDL-transferred microRNA-223 regulates ICAM-1 expression in endothelial cells. Nat Commun 5, 3292–3303 (2014). 24576947 10.1038/ncomms4292 PMC4189962

[b34] Menghini R. *et al.* MicroRNA 217 Modulates Endothelial Cell Senescence via Silent Information Regulator 1. Circulation 120, 1524–1532 (2009). 19786632 10.1161/CIRCULATIONAHA.109.864629

[b35] Donners M. M. *et al.* Hematopoietic miR155 deficiency enhances atherosclerosis and decreases plaque stability in hyperlipidemic mice. PLoS One 7, e35877 (2012). 22558252 10.1371/journal.pone.0035877 PMC3338496

[b36] Ceppi M. *et al.* MicroRNA-155 modulates the interleukin-1 signaling pathway in activated human monocyte-derived dendritic cells. Proc Natl Acad Sci USA 106, 2735–2740 (2009). 19193853 10.1073/pnas.0811073106 PMC2650335

[b37] Zhu N. *et al.* Endothelial enriched microRNAs regulate angiotensin II-induced endothelial inflammation and migration. Atherosclerosis 215, 286–293 (2011). 21310411 10.1016/j.atherosclerosis.2010.12.024

[b38] Wu X., Fan W., Fang R. & Wu G. Regulation of microRNA-155 in endothelial inflammation by targeting nuclear factor (NF)-κB P65. J Cell Biochem 115, 1928–1936 (2014). 24905663 10.1002/jcb.24864

[b39] Huang R. S., Hu G. Q., Lin B., Lin Z. Y. & Sun C. C. MicroRNA-155 Silencing Enhances Inflammatory Response and Lipid Uptake in Oxidized Low-Density LipoproteinYStimulated Human THP-1 Macrophages. J Investig Med 58, 961–967 (2010). 10.231/JIM.0b013e3181ff46d7 21030878

[b40] Sun H. X. *et al.* Essential Role of MicroRNA-155 in Regulating Endothelium-Dependent Vasorelaxation by Targeting Endothelial Nitric Oxide Synthase. Hypertension 60, 1407–1414 (2012). 23108656 10.1161/HYPERTENSIONAHA.112.197301

[b41] Zhang R. N. *et al.* Tongxinluo inhibits vascular inflammation and neointimal hyperplasia through blockade of the positive feedback loop between miR-155 and TNF-α. AJP: Heart and Circulatory Physiology 307, H552–562 (2014). 24951754 10.1152/ajpheart.00936.2013

[b42] Tian F. J. *et al.* Elevated microRNA-155 promotes foam cell formation by targeting HBP1 in atherogenesis. Cardiovasc Res 103, 100–110 (2014). 24675724 10.1093/cvr/cvu070

[b43] Groblewski G. E. *et al.* Purification and characterization of a novel physiological substrate for calcineurin in mammalian cells. J Biol Chem 273, 22738–22744 (1998). 9712905 10.1074/jbc.273.35.22738

[b44] Hou H. *et al.* Structure-functional analyses of CRHSP-24 plasticity and dynamics in oxidative stress response. J Biol Chem 286, 9623–9635 (2011). 21177848 10.1074/jbc.M110.177436 PMC3058955

[b45] Vargova K. *et al.* MYB transcriptionally regulates the miR-155 host gene in chronic lymphocytic leukemia. Blood 117, 3816–3825 (2011). 21296997 10.1182/blood-2010-05-285064

[b46] Yin Q., Wang X., McBride J., Fewell C. & Flemington E. B-cell Receptor Activation Induces BIC/miR-155 Expression through a Conserved AP-1 Element. J Biol Chem 283, 2654–2662 (2008). 18048365 10.1074/jbc.M708218200 PMC2810639

[b47] Li P. *et al.* Signal transducer and activator of transcription-3 induces microRNA-155 expression in chronic lymphocytic leukemia. PLoS One 8 e64678 (2013). 23750211 10.1371/journal.pone.0064678 PMC3672147

[b48] Thompson R. C., Vardinogiannis I. & Gilmore T. D. Identification of an NF-kappaB p50/p65-responsive site in the human MIR155HG promoter. BMC Mol Biol 14, 24 (2013). 24059932 10.1186/1471-2199-14-24 PMC3849010

[b49] Gatto G. *et al.* Epstein-Barr virus latent membrane protein 1 trans-activates miR-155 transcription through the NF- B pathway. Nucleic Acids Res 36, 6608–6619 (2008). 18940871 10.1093/nar/gkn666 PMC2582607

[b50] Daigneault M., Preston J. A., Marriott H. M., Whyte M. K. & Dockrell D. H. The identification of markers of macrophage differentiation in PMA-stimulated THP-1 cells and monocyte-derived macrophages. PLoS One 5, e8668 (2010). 20084270 10.1371/journal.pone.0008668 PMC2800192

[b51] Bao Y. *et al.* Salvianolic acid B inhibits macrophage uptake of modified low density lipoprotein (mLDL) in a scavenger receptor CD36-dependent manner. Atherosclerosis 223, 152–159 (2012). 22658257 10.1016/j.atherosclerosis.2012.05.006 PMC3389144

[b52] Schmittgen T. D. & Livak K. J. Analyzing real-time PCR data by the comparative C(T) method. Nat Protoc 3, 1101–1108 (2008). 18546601 10.1038/nprot.2008.73

[b53] Subbaram S. *et al.* Integrin 3 1 controls mRNA splicing that determines Cox-2 mRNA stability in breast cancer cells. J Cell Sci 127, 1179–1189 (2014). 24434582 10.1242/jcs.131227 PMC3953813

[b54] Xu T. P. *et al.* SP1-induced upregulation of the long noncoding RNA TINCR regulates cell proliferation and apoptosis by affecting KLF2 mRNA stability in gastric cancer. Oncogene 34, 5648–5661 (2015). 25728677 10.1038/onc.2015.18

