# Supplementary material for: miR-155 acts as an anti-inflammatory factor in atherosclerosis-associated foam cell formation by repressing calcium-regulated heat stable protein 1
Source: Sci Rep. 2016 Feb 22;6:21789. doi: 10.1038/srep21789 (PMC4761895; doi:10.1038/srep21789)

Supplementary materials

miR-155 acts as an anti-inflammatory factor in atherosclerosis-associated foam cell formation by repressing calcium-regulated heat stable protein 1

Xiaoyi Li[a,b1], Deyong Kong[a1], Heming Chen[c1], Shuiyi Liu[a,d], Hui Hu[a], Tangwei Wu[a], Jing Wang[a], Weiqun Chen[a,d,e], Yong Ning[b], Yong Li[a,f*], Zhongxin Lu[a,b,d*]

[a] Department of Medical Laboratory, Central Hospital of Wuhan, Wuhan 430014, China

[b] School of Laboratory Medicine, Hubei University of Chinese Medicine, Wuhan 430065, China

[c] Department of Cardiovascular and Thoracic Surgery, Second Xiangya Hospital, Central South University, Changsha 410011, China

[d] Cancer Research Institute of Wuhan, Wuhan 430014, China

[e] Department of Central Laboratory, Central Hospital of Wuhan, Wuhan 430014, China

[f] Department of Cancer Biology, Lerner Research Institute, Cleveland Clinic, Cleveland, OH 44195, USA

Supplementary Figure S1： Full-length blots for Figure 3D

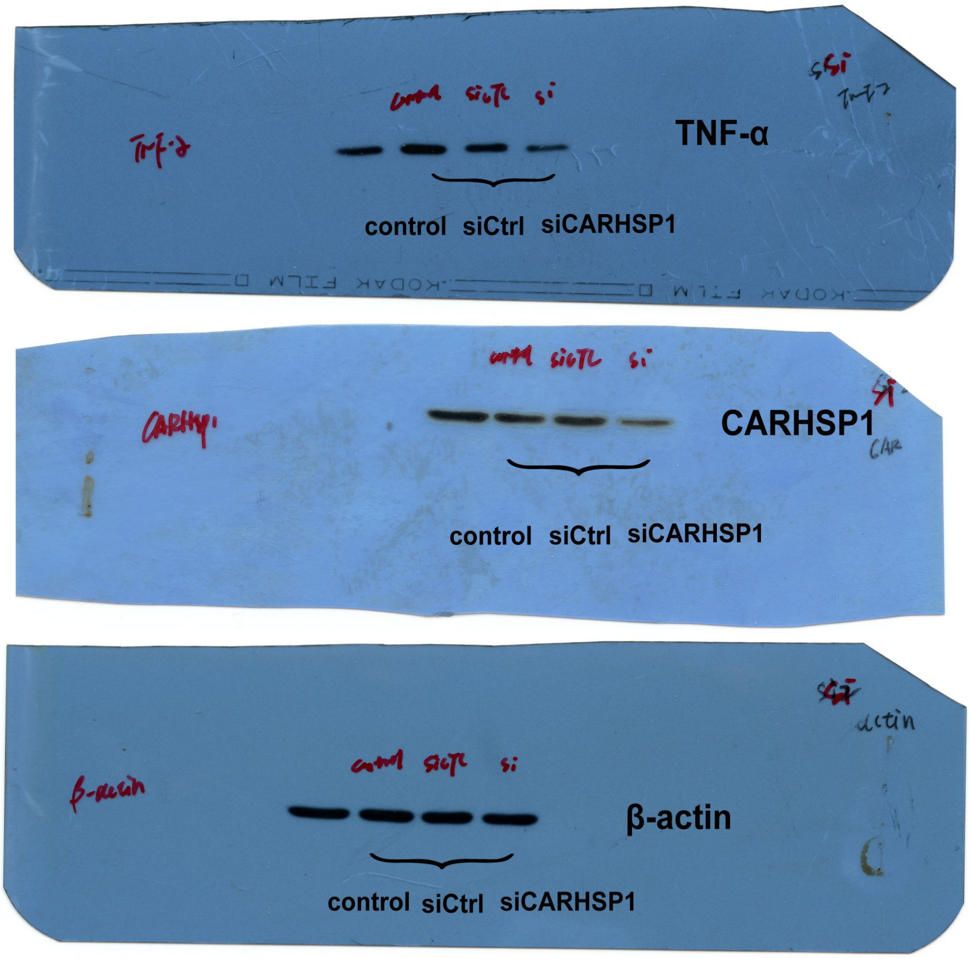

Supplementary Figure S2： Full-length blots for Figure 3H

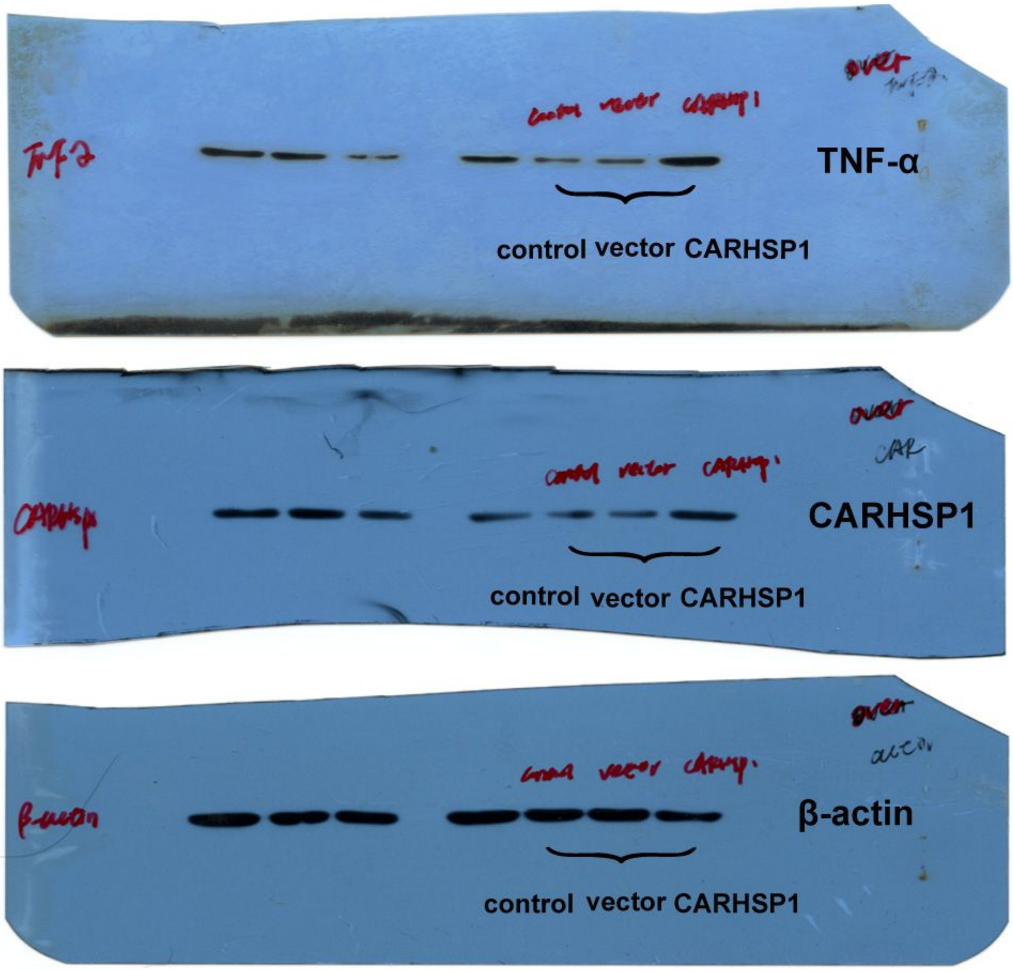

Supplementary Figure S3： The conservation of the binding site of miR-155 in CARHSP1

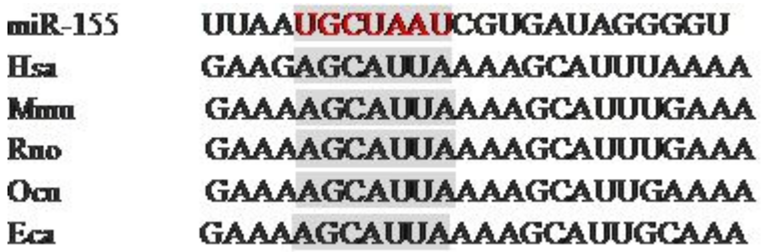

Figure S3. The nucleotides complementary to the miR-155 seed sequences are highly conserved in human, mouse, rat, rabbit and horse. he miR-155 seed sequeces are in red and the complementary nucleotides in the 3'UTR of CARHSP1 are gray shade.

Supplementary Figure S4： Full-length blots for Figure 4D

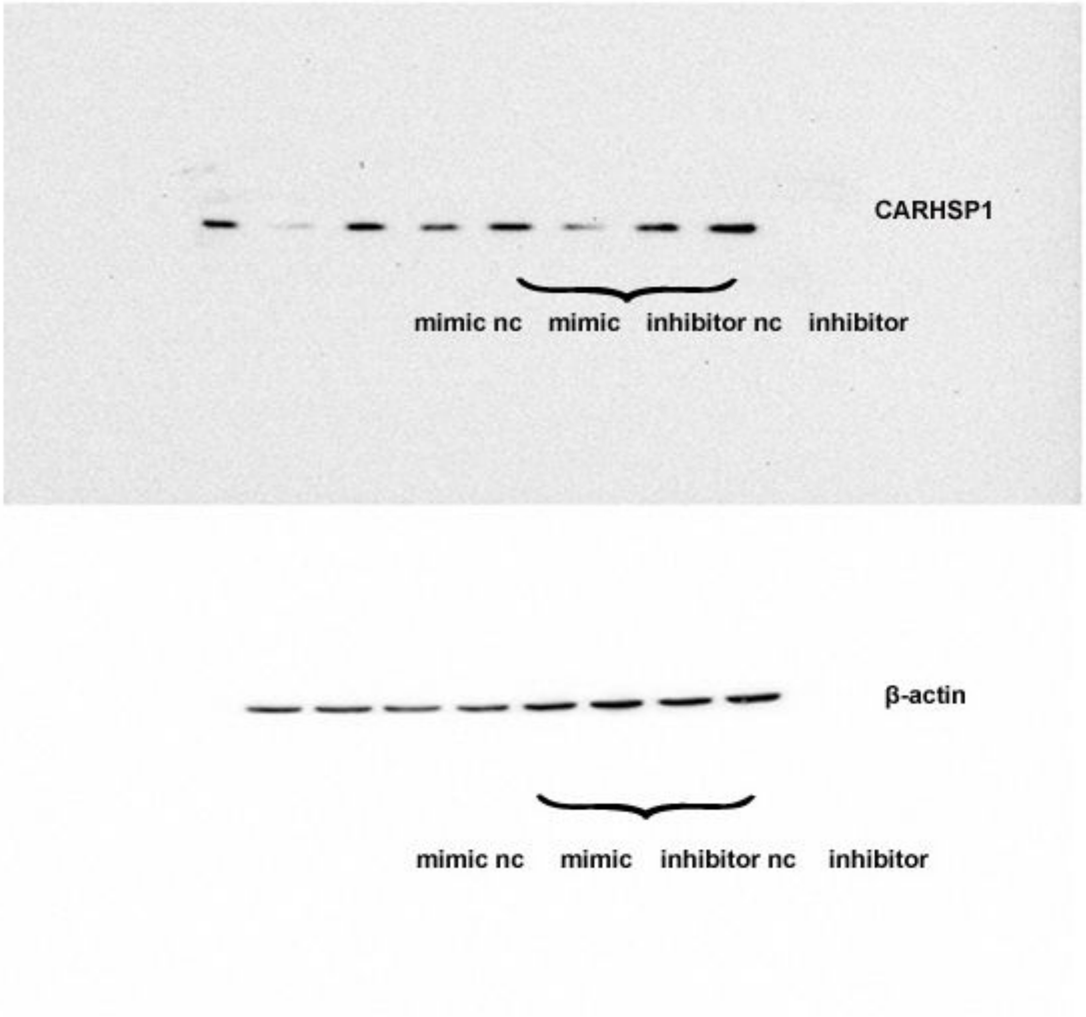

Supplementary Figure S5: Full-length blots for Figure 4H

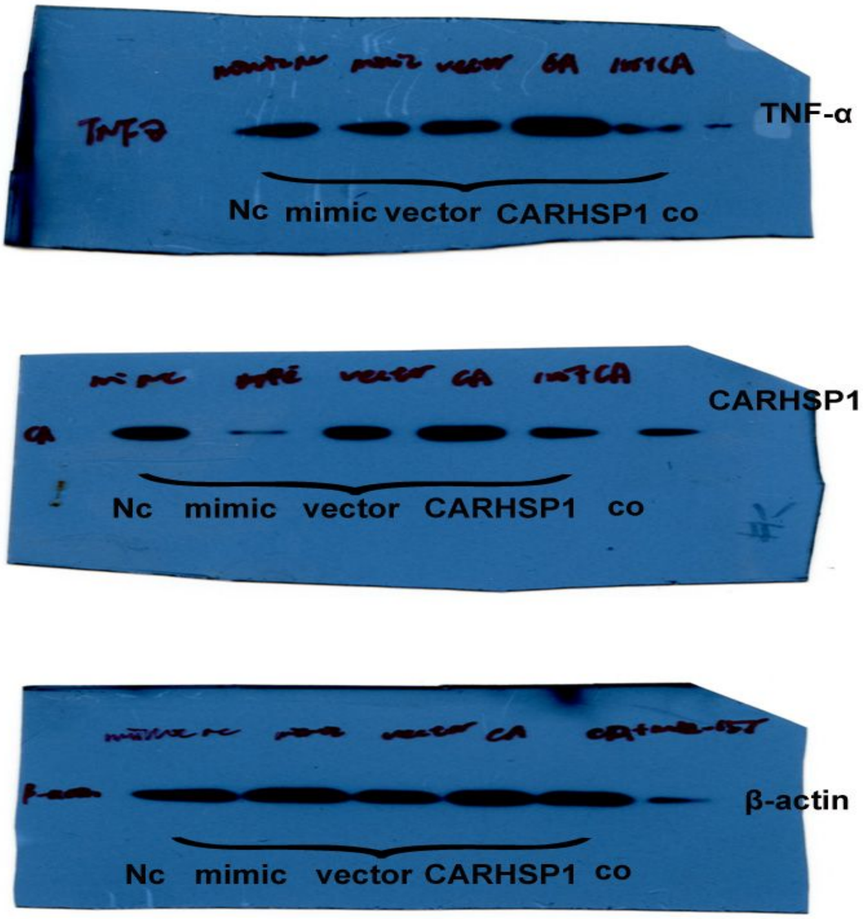

Supplementary Figure S6： Full-length blots for Figure 5B

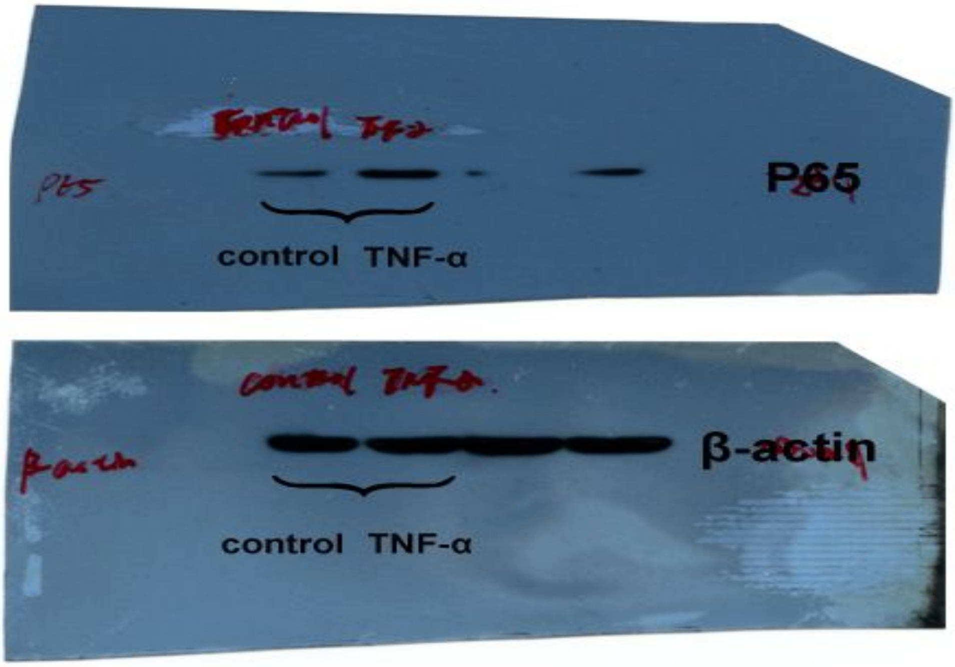

Supplementary Figure S7： Full-length gels for Figure 5C

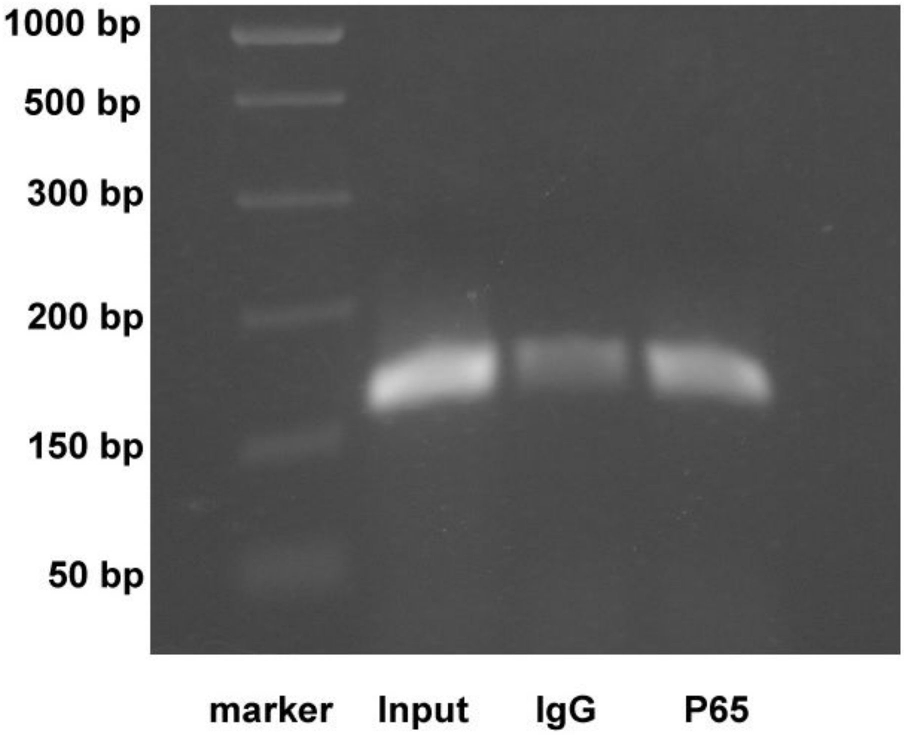

Supplement: Supplementary Information [file srep21789-s1.pdf]
